# Any increment in physical activity reduces mortality risk of physically inactive patients: prospective cohort study in primary care

**DOI:** 10.3399/BJGP.2022.0118

**Published:** 2022-11-01

**Authors:** Gonzalo Grandes, Arturo García-Alvarez, Maider Ansorena, Ricardo Ortega Sánchez-Pinilla, Jesus Torcal, María Soledad Arietaleanizbeaskoa, Alvaro Sánchez

**Affiliations:** Head of the primary care research unit of Bizkaia; Primary Care Research Unit of Bizkaia, Osakidetza (Basque Healthcare Service), BioCruces-Bizkaia Health Research Institute, Bilbao, Spain.; Primary Care Research Unit of Bizkaia, Osakidetza (Basque Healthcare Service), BioCruces-Bizkaia Health Research Institute, Bilbao, Spain.; Santa Barbara Primary Care Centre, Castilla-La Mancha Health Service, Toledo, Spain.; Primary Care Research Unit of Bizkaia, Osakidetza (Basque Healthcare Service), BioCruces-Bizkaia Health Research Institute, Bilbao, Spain.; Primary Care Research Unit of Bizkaia, Osakidetza (Basque Healthcare Service), BioCruces-Bizkaia Health Research Institute, Bilbao, Spain.; Primary Care Research Unit of Bizkaia, Osakidetza (Basque Healthcare Service), BioCruces-Bizkaia Health Research Institute, Bilbao, Spain.

**Keywords:** cohort studies, exercise, health behaviour, mortality, primary health care, survival analysis

## Abstract

**Background:**

It is unclear how engaging in physical activity after long periods of inactivity provides expected health benefits.

**Aim:**

To determine whether physically inactive primary care patients reduce their mortality risk by increasing physical activity, even in low doses.

**Design and setting:**

Prospective cohort of 3357 physically inactive patients attending 11 Spanish public primary healthcare centres.

**Method:**

Change in physical activity was repeatedly measured during patients’ participation in the ‘Experimental Program for Physical Activity Promotion’ clinical trial between 2003 and 2006, using the ‘7-day Physical Activity Recall’. Mortality to 31 December 2018 (312 deaths) was recorded from national statistics, and survival time from the end of the clinical trial analysed using proportional hazard models.

**Results:**

After 46 191 person–years of follow-up, compared with individuals who remained physically inactive, the mortality rates of those who achieved the minimum recommendations of 150– 300 min/ week of moderate- or 75–150 min/ week of vigorous-intensity exercise was reduced by 45% (adjusted hazard ratio [aHR] 0.55; 95% confidence interval [95% CI] = 0.41 to 0.74); those who did not meet these recommendations but increased physical activity in low doses, that is, 50 min/week of moderate physical activity, showed a 31% reduced mortality (aHR 0.69, 95% CI = 0.51 to 0.93); and, those who surpassed the recommendation saw a 49% reduction in mortality (aHR 0.51, 95% CI = 0.32 to 0.81). The inverse association between increased physical activity and mortality follows a continuous curvilinear dose–response relationship.

**Conclusion:**

Physically inactive primary care patients reduced their risk of mortality by increasing physical activity, even in doses below recommended levels. Greater reduction was achieved through meeting physical activity recommendations or adopting levels of physical activity higher than those recommended.

## INTRODUCTION

The benefits of physical activity are unquestionable, and previous studies estimate that mortality rates in people who are active are reduced by between 30% and 60% compared with those who are not active.^1^^–^5 Despite this, it is estimated that worldwide, 27.5% of adults and 81% of adolescents do not meet the minimum recommendations of 150 min/week of moderate physical activity or 75 min/week of vigorous activity, and in more developed countries these recommendations are not met by >40% of the population.^6^^–^10 Focusing specifically on the primary care consulting population, the proportion of patients who are inactive is even higher, close to 70% in Spain,^11^ and as inactivity is one of the most important risk factors for health,^12^ primary care professionals often try to promote physical activity to their patients through interventions of proven effectiveness.^13^^,^^14^ However, it is unclear whether primary care patients who have spent much of their lives being inactive could experience benefits associated with physical activity by becoming more active, and whether shifting from no activity to minimal increases in physical activity translates into health benefits and substantial impact on mortality.

Some longitudinal studies have tried to clarify this uncertainty, although they are scarce and, to the authors’ knowledge, they are based on cohorts of convenience samples of the general population or subgroups of patients with specific pathologies, rather than being based on the whole population of patients attending primary care.^15^^–^23 What is needed, therefore, are long-term longitudinal studies following inactive primary care patients where a change in the level of physical activity over time is measured to analyse if and how this change is associated with mortality. The objective of this study was to evaluate the effect on mortality that engagement in physical activity has on a previously inactive primary care population, even when there are only modest increases in physical activity below current recommendations.

## METHOD

This was a cohort study of 3357 primary care patients who were inactive and who participated in the PEPAF (Experimental Program for Physical Activity Promotion from ‘Programa Experimental de Promoción de la Actividad Fisica’, in Spanish) clinical trial and who did not meet current recommended levels of physical activity. The participants were from 11 primary healthcare centres in eight Spanish autonomous regions and aged between 19 and 80 years. Patients were recruited through systematic sampling of all patients scheduled for consultation with physicians following a random procedure.^11^

**Table:** How this fits in

People who are physically active show a reduced risk of premature mortality. Can primary care patients, after being inactive for much of their lives, reduce their mortality risk when they shift from no activity to any increment in physical activity? This research found that patients who were inactive but increased their physical activity even at a minimal level, below minimum guideline recommendations, significantly reduced their mortality risk, and those who adopted current recommendations or an even higher level of activity had a lower mortality risk in a curvilinear dose– response relationship. These findings should encourage primary healthcare professionals to promote physical activity and facilitate initial negotiation of objectives when prescribing a physical activity plan.

Their level of physical activity was repeatedly measured between 2003 and 2006, at 6, 12, and 24 months after their inclusion in the clinical trial.^24^ Subsequently, their mortality was recorded, after 12 years, as of 31 December 2018, using data provided by the National Institute of Statistics of Spain, and survival time was calculated from the last physical activity measurement in 2006.

### Exposure measurement

Change in physical activity was measured throughout the 24-month follow-up of the participants in the PEPAF clinical trial using the semi-structured 7-day Physical Activity Recall (PAR) interview, which has highly accredited validity and reliability.^25^^,^^26^ The PAR counts the episodes of physical activity that last >10 min in the 7 days before the interview, and is able to calculate compliance with the physical activity recommendations and the activity dose in metabolic equivalent of task (MET) × h/day, multiplying the hours dedicated to activities of moderate, vigorous, and very vigorous intensity by the corresponding METs: 4, 6, and 10, respectively.

### Covariates

In the PEPAF clinical trial, abdominal girth, body mass index, and the percentage of body fat based on the thickness of cutaneous folds were measured following the protocols of the American College of Sports Medicine (ACSM).^27^ Active health problems in the 12 months before the start of the study, subsequently grouped with the Ambulatory Care Groups (ACGs) case mix system,^28^ and cardiovascular risk factors at study entry were reported by family doctors after reviewing the medical history. Additionally, research nurses carried out the measurement of alcohol consumption (AUDIT),^29^ tobacco consumption, blood pressure, cholesterol, blood glucose, blood pressure, educational level, and social class according to the recommendations of the Spanish Epidemiology Society.^30^

### Statistical analysis

Cox proportional hazards models were used to estimate adjusted hazard ratios (aHRs) of mortality with 95% confidence intervals (95% CIs). Average increment of physical activity reported by participants at 6, 12, and 24 months after baseline measurement, weighted by time elapsed from the previous assessment, assigning a zero for missing values in single-time measurements because of patient non-attendance as the most likely outcome in patients who are inactive and the most conservative way of handling them, were grouped into four exposure groups that match leisure time physical activity (LTPA) doses with physical activity guidelines:^6^

- reference group including those who did not increment LTPA at all;
- the low-dose category, which included those who increased their LTPA but did not meet current minimum recommendations of 150 min/week of moderate-intensity or 75 min/week of vigorous-intensity (<1 MET × h/day) activity;
- those who met recommendations of 150–300 min/week of moderate- or 75–150 min/week of vigorous-intensity (1–3.9 MET × h/day) activity; and
- the very high-dose category, including those who surpassed the recommended activity doses (≥4 MET × h/day).

Statistical models were simultaneously adjusted for known factors associated with mortality and potential confounding variables, such as baseline age, sex, socioeconomic status, education level, smoking, dyslipidaemia, blood pressure, obesity, alcohol consumption, diabetes, cancer, cardiovascular disease, other chronic conditions, and primary care centre, based on the causal directed acyclic graph shown in Supplementary Figure S1.^31^ Backward, forward, and stepwise selection strategies were used and likelihood ratio tests performed with a 0.05 significance level. The potential modification of the effect of LTPA by age and sex was evaluated by testing first and second grade interaction terms using a significance threshold of 0.01.

The analysis was repeated considering LTPA as a continuous variable following a linear, quadratic, or cubic function, and different transformations. Goodness-of-fit tests and their appropriate semi-parabolic shape led the authors to select the square root transformation. Proportional hazard assumption was checked, using log–log plots, statistical tests for the interaction of time with the variables, and Schoenfeld residual plots, showing no significant violations. Participants who died and survivors were compared using logistic regression analysis to check the correct identification of all the variables associated with mortality to be included in the proportional hazards models. Sensitivity analyses were performed by excluding those who died within the first, second, and third year and by using a two-part regression model (accounting for the excess of zeros) imputation method for the physical activity missing values. The authors estimated the proportion of avoidable deaths attributable to physical inactivity among the entire population, that is, the population attributable fraction.^32^ Statistical analyses were performed using SAS (version 9.4) and R (version 3.5.1) software.

## RESULTS

Of the 3357 primary care attendees who did not meet the minimum physical activity recommendations at baseline:

- 1031 (30.7%) did not report any LTPA increment over the 2-year follow-up in the PEPAF clinical trial;
- 917 (27.3%) reported a 2-year LTPA average increment <1 MET × h/day;
- 1190 (35.4%) reported an increase of between 1–3.9 MET, which corresponds with meeting the current physical activity recommendations; and
- 219 (6.5%) reported an average increment that was ≥4 MET × h/day (Table 1).

**Table 1.** All-cause mortality associated with physical activity increment and baseline characteristics of inactive primary care attendees

**Category**	***n***	**Person–years**	**Deaths, *n***	**Adjusted hazard ratio (95% CI)^a^**
2-year average LTPA increment (MET*h/d)				
Null (=0)	1031	14 117.3	106	1 (reference)
Low doses (<1)	917	12 697.9	78	0.69 (0.51 to 0.93)
Meet recommendations (1–3.9)	1190	16 398.6	103	0.55 (0.41 to 0.74)
Very high doses (≥4)	219	2977.3	25	0.51 (0.32 to 0.81)

**Baseline characteristics**				
Age (each year)	—	—	—	1.12 (1.10 to 1.13)
Sex				
Female	2202	30 819.5	136	1 (reference)
Male	1155	15 371.6	176	1.63 (1.22 to 2.18)
Smoking status				
Never smoker	1741	24 036.5	156	1 (reference)
Ex-smoker	635	8509.5	87	1.37 (1.0 to 1.90)
Current smoker	981	13 645.0	69	1.58 (1.13 to 2.22)
Blood pressure				
Normal	1605	22 674.3	70	1 (reference)
High-normal	641	8730.5	72	1.56 (1.11 to 2.18)
Grade I hypertension	845	11 334.8	110	1.41 (1.02 to 1.96)
Grade II hypertension	266	3451.6	60	1.77 (1.21 to 2.60)
Diabetes				
No	3075	42 657.0	242	1 (reference)
Yes	282	3534.1	70	1.37 (1.04 to 1.81)
Cancer				
No	3287	45 399.5	288	1 (reference)
Yes	70	791.6	24	3.48 (2.25 to 5.37)
Chronic unstable disease				
No	3047	42 399.8	216	1 (reference)
Yes (ADG11)	310	3791.4	96	2.56 (1.98 to 3.32)
Psychosocial condition				
No	2648	36 510.8	235	1 (reference)
Yes (CADG10)	709	9680.4	77	1.53 (1.17 to 2.00)

a *Simutaneously adjusted for all the above variables as well as for the primary care center. ADG11 and CADG10: case-mix categories of the Ambulatory Care Groups patients’ classification system. Primary care center* P *= 0.002. LTPA = leisure time physical activity. MET = metabolic equivalent of task.*

All of the patients had at least one physical activity measurement (at 6, 12, or 24 months) recorded, 3001 (89.4%) at least two, and 2506 (74.6%) all three. Of the 10 071 total expected measurements, 1207 (12.0%) were missed, and from those conducted 4484 (50.6%) had a value of 0 MET × h/day (data not shown).

Patient baseline characteristics are shown in Supplementary Table S1. They were predominantly female (65.6%), with a mean age of 50.7 years (standard deviation [SD] 14.8, range 19–80), and mostly manual workers without a university education. The characteristics of the participants were not well balanced between the four LTPA comparison groups. Females, younger adults (average age 49 versus 52 years), those with a lower educational level, lower social class, and those who had cancer and cardiovascular risk factors were more likely not to increment LTPA whereas those with other chronic comorbidities at baseline were more likely to increment their LTPA level over the 2-year follow-up in the PEPAF clinical trial. During the 12-year follow-up after finishing the PEPAF clinical trial, 312 participants died, most of them because of cancer ( *n* = 130, 41.7%), 63 (20.2%) from cardiovascular disease, 34 (10.9%) from respiratory diseases, and 20 (6.4%) from neurological disorders, finally, 65 people (20.8%) died from other causes.

Supplementary Table S2 shows the characteristics associated with mortality, such as male sex, older age, low level of education and social class, obesity, diabetes, other risk factors, and chronic diseases. The aforementioned crude associations between participants’ characteristics, LTPA, and mortality highlight the need for adjustment by all these potential confounders to estimate the causal effect of LTPA increment on mortality.

After adjustment for sociodemographic characteristics, baseline risk factors, and chronic diseases, compared with individuals in the null LTPA increment group, those who increased daily LTPA <1 MET × h/day had a 31% reduced risk for all-cause mortality (aHR 0.69, 95% CI = 0.51 to 0.93); those who increased LTPA to meet guideline recommendations experienced reduced mortality by 45% (aHR 0.55; 95% CI = 0.41 to 0.74); and those who surpassed these recommendations, increasing LTPA by ≥4 MET × h/day, experienced the highest mortality reduction, by approximately 50% (aHR 0.51, 95% CI = 0.32 to 0.81) (Table 1).

**Figure 1.**
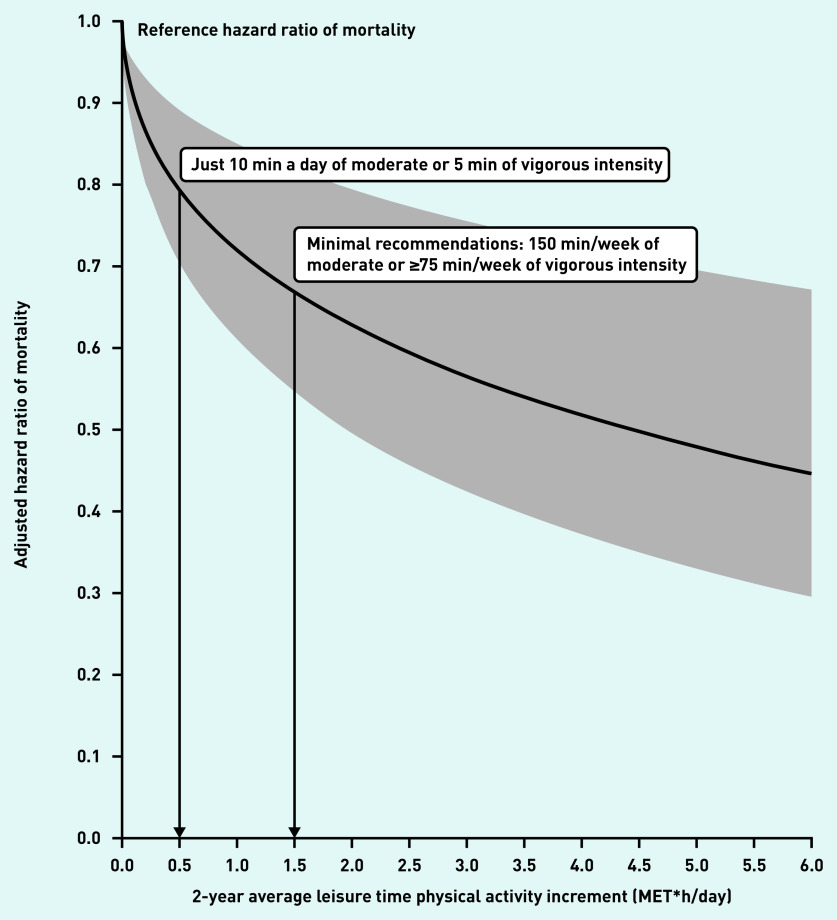
*All-cause mortality reduction associated with physical activity increment in previously inactive primary care attendees. Leisure time physical activity (LTPA) as a continuous quantitative variable with square root transformation. MET = metabolic equivalent of task.*

Figure 1 shows the reductions in all-cause mortality associated with daily LTPA compared with those individuals who did not increase LTPA at all. When analysing the dose– response relationship between mortality and LTPA considered quantitatively, results were consistent with the aforementioned categorical analysis, showing a steep reduction in mortality risk, close to 20%, associated with a minimal LTPA increment of 0.5 MET × h/ day (equivalent to 10 min of moderate LTPA a day), followed by a progressive drop to an approximate 45% decrease in mortality at 3 MET × h/day (equivalent to 1 h of moderate LTPA or 25 min of vigorous LTPA a day), with further positive effects on mortality as LTPA incremented. Neither significant nor meaningful modifications of the effect of LTPA on mortality in the age and sex subgroups ( *P*>0.28) was found (data not shown).

With an estimated aHR of 1.51 for those not meeting minimum physical activity recommendations (95% CI = 1.19 to 1.92), the population fraction of deaths attributable to inactivity is 20% (95% CI = 9.4 to 28.3), that is, 62 out of 312 deaths that occurred in the study population during the 12-year follow-up would have been avoided if the entire population had adopted the minimum recommendations (data not shown).

Consistent with the reported survival analyses, logistic regression analyses examining the probability of dying found a 32% reduction in the odds of death associated with the minimal increments in LTPA (adjusted odds ratio [aOR] 0.68, 95% CI = 0.46 to 0.99), a 50% reduction associated with meeting the current physical activity recommendations (aOR 0.50, 95% CI = 0.35 to 0.71), and a 52% reduction for those who surpassed these recommendations (aOR 0.48, 95% CI = 0.26 to 0.87) (data not shown).

Restricting the survival analyses to deaths that occurred after the first, second, and third year of follow-up did not show meaningful differences in the reported results (aHR for the low-dose group <1 MET × h/day 0.70, 95% CI = 0.51 to 0.96; aHR for those meeting recommendations of 1–3.9 MET × h/day 0.52, 95% CI = 0.38 to 0.71; and aHR for the very high-dose group ≥4 MET × h/day 0.46, 95% CI = 0.27 to 0.76; when restricting analyses to deaths after the third year of follow-up). There were also not significant differences when the missing values were imputed for physical activity using the two-part regression model (aHR for low-dose group <1 MET × h/day 0.68, 95% CI = 0.49 to 0.93; aHR for those meeting recommendations of 1–3.9 MET × h/day 0.56, 95% CI = 0.42 to 0.76; and aHR for the very high-dose group ≥4 MET × h/day 0.54, 95% CI = 0.34 to 0.85) (data not shown).

## DISCUSSION

### Summary

The results in the present study confirm that an increase in physical activity levels in an inactive population of primary care patients translates into a significant reduction in mortality. These benefits follow a clear dose–response relationship, in which mortality starts to fall even with only small increases in physical activity, for example, 10 min of moderate activity per day or 50 min per week, and mortality continues to decrease progressively, so that the risk of death is approximately halved when the minimum recommendations regarding activity are met. This has been observed in a population that was inactive, probably for many years before entering this study, after 46 191 person–years of follow-up, and, if these recommendations had been met by the whole population, it would have prevented one in every five deaths observed in this study, an impact on mortality equal to or greater than that attributed to other risk factors, such as tobacco, hypertension, obesity, or diabetes.^12^

Although physical activity promotion by healthcare professionals is a highly efficient intervention (with a number needed to treat of 12),^13^ the minimum recommended threshold of 150 min/week of moderate or 75 min/week of vigorous physical activity may be perceived as a barrier for patients who have been inactive long term. As a result, the finding in the current study that minimal increases in the level of physical activity, even below the minimum recommendations established by international organisations, observed in an inactive population of patients are associated with a significant reduction in mortality is very important.

When primary healthcare professionals try to promote physical activity in patients who have been inactive for many years, it is easier to negotiate a small initial goal, such as including 10 min of moderate activity a day, which will benefit them and can later be increased progressively. This applies equally to males and females in all age groups, who in the current study, benefit equally from an increase in their levels of physical activity. This makes it a universal health promotion intervention for primary care that achieves significant health benefits, even with the most modest of increments in physical activity.

### Strengths and limitations

To the authors’ knowledge, this study is one of the first to explicitly evaluate the effect of increasing physical activity, measured over 2 years, in a large representative sample of primary care patients who are inactive. Although objective measures of physical activity levels are desirable, to avoid recall and social desirability bias, the use of structured self-reported measurements are an accepted and extensively used method in population-based epidemiological studies linking physical activity and health. Nevertheless, the 7-day PAR interview has shown a good correlation with objective measures of physical activity and considering that all study participants performed the same measurements with certified nurses, measurement error is expected to be non-differential among the exposure groups.^26^ Missing values in some of the single-time physical activity measurements may also introduce bias and threaten the validity of results. However, the authors consider this bias to be negligible as the different imputation methods conducted, both assigning a value of zero activity (being the most likely value in the repeated measurements) and using the two-part regression model, provided similar results. The observational nature of the study does not preclude the possibility of residual confounding, although a significant number of possible confounding factors have been controlled for. Although sensitivity analyses excluding deaths within the first 2 and 3 years of follow-up reduce the likelihood of reverse causality owing to undetected hidden diseases at baseline, it is not possible to completely rule this out.

### Comparison with existing literature

To the authors’ knowledge, the present study is the only one that has been carried out in primary care with males and females of all ages who are inactive and studying reduction in mortality associated with a change from being inactive to active. Previously, Wannamethee *et al* published a study in primary care in which only males participated and the results are consistent with the results in the present study.^15^ Three other previous studies that have looked into the decrease in mortality in a primary care population associated with compliance with minimum recommendations for activity were carried out in selected subgroups, and their results are consistent with the decrease in mortality shown in the present study when the minimum recommendations of activity are met.^17^^,^^21^^,^^22^ In this regard, the present study also includes the fact that a 31% decrease in mortality is observed even without reaching these minimum recommendations of activity. This finding is consistent with the results of previous epidemiological studies that have shown that a minimum amount of physical activity reduces mortality.^2^^,^^33^^,^^34^

### Implications for research and practice

The potential public health impact of promoting physical activity in this primary care population is an estimated 20% of deaths that could have been avoided if all participants had adopted the minimum recommendations of accumulating at least 150 min/week of moderate- or 75 min/ week of vigorous-intensity activity. This study also shows that even without reaching this minimum level of activity, the impact of increased activity on mortality is very important for males and females in all age groups. The message should be that any increase in physical activity in patients who are inactive is clearly better than nothing, although the greater the activity, the greater the benefits. Additionally, as other studies have shown, with respect to intensity, low-intensity physical activity also produces substantial benefits,^35^ although more benefits could be obtained at moderate intensity, and increasing vigorous-intensity physical activity is associated with additional health benefits.^36^

In conclusion, ‘exercise is medicine’: encouraging primary care patients to abandon inactivity and adopt any amount of physical activity, even below the threshold of the guidelines’ recommendations, reduces all-cause mortality preventing a substantial number of deaths. Physical activity assessment and promotion in routine clinical practice is an effective and efficient medicine for helping patients to become healthier and for extending longevity. Innovative implementation strategies are needed to translate proven physical activity promotion interventions into practice in a sustainable and generalisable way.^37^

## Data

Data available to researchers on request.

## References

[1] Paffenbarger RS, Hyde RT, Wing AL (1986). Physical activity, all-cause mortality, and longevity of college alumni. N Engl J Med.

[2] Wen CP, Wai JPM, Tsai MK (2011). Minimum amount of physical activity for reduced mortality and extended life expectancy: a prospective cohort study. Lancet.

[3] Arem H, Moore SC, Patel A (2015). Leisure time physical activity and mortality: a detailed pooled analysis of the dose-response relationship. JAMA Intern Med.

[4] Hupin D, Edouard P, Gremeaux V (2017). Physical activity to reduce mortality risk. Eur Heart J.

[5] Department of Health and Human Sciences (2018). 2018 physical activity guidelines advisory committee scientific report.

[6] Bull FC, Al-Ansari SS, Biddle S (2020). World Health Organization 2020 guidelines on physical activity and sedentary behaviour. Br J Sports Med.

[7] Guthold R, Stevens GA, Riley LM, Bull FC (2018). Worldwide trends in insufficient physical activity from 2001 to 2016: a pooled analysis of 358 population-based surveys with 1.9 million participants. Lancet Glob Health.

[8] Guthold R, Stevens GA, Riley LM, Bull FC (2020). Global trends in insufficient physical activity among adolescents: a pooled analysis of 298 population-based surveys with 1·6 million participants. Lancet Child Adolesc Health.

[9] Pereira JR, Cliff DP, Sousa-Sá E (2019). Prevalence of objectively measured sedentary behavior in early years: systematic review and meta-analysis. Scand J Med Sci Sports.

[10] Marques A, Henriques-Neto D, Peralta M (2020). Prevalence of physical activity among adolescents from 105 low, middle, and high-income countries. Int J Environ Res Public Health.

[11] Grandes G, Sánchez A, Torcal J (2008). Targeting physical activity promotion in general practice: characteristics of inactive patients and willingness to change. BMC Public Health.

[12] Murray CJL, Aravkin AY, Zheng P (2020). Global burden of 87 risk factors in 204 countries and territories, 1990–2019: a systematic analysis for the Global Burden of Disease Study 2019. Lancet.

[13] Orrow G, Kinmonth AL, Sanderson S, Sutton S (2012). Effectiveness of physical activity promotion based in primary care: systematic review and meta-analysis of randomised controlled trials. BMJ.

[14] Kettle VE, Madigan CD, Coombe A (2022). Effectiveness of physical activity interventions delivered or prompted by health professionals in primary care settings: systematic review and meta-analysis of randomised controlled trials. BMJ.

[15] Wannamethee SG, Shaper AG, Walker M (1998). Changes in physical activity, mortality, and incidence of coronary heart disease in older men. Lancet.

[16] Steffen-Batey I, Nichaman MZ, Goff DC (2000). Change in level of physical activity and risk of all-cause mortality or reinfarction: the Corpus Christi heart project. Circulation.

[17] Mok A, Khaw KT, Luben R (2019). Physical activity trajectories and mortality: population based cohort study. BMJ.

[18] Byberg L, Melhus H, Gedeborg R (2009). Total mortality after changes in leisure time physical activity in 50 year old men: 35 year follow-up of population based cohort. BMJ.

[19] Gregg EW (2003). Relationship of changes in physical activity and mortality among older women. JAMA.

[20] Petersen Christina Bjørk (2012). Changes in physical activity in leisure time and the risk of myocardial infarction, ischemic heart disease, and all-cause mortality. Eur J Epidemiol.

[21] Gorczyca AM, Eaton CB, LaMonte MJ (2017). Change in physical activity and sitting time after myocardial infarction and mortality among postmenopausal women in the Women’s Health Initiative-Observational Study. J Am Heart Assoc.

[22] Holme I, Anderssen SA (2015). Increases in physical activity is as important as smoking cessation for reduction in total mortality in elderly men: 12 years of follow-up of the Oslo II study. Br J Sports Med.

[23] Talbot LA, Morrell CH, Fleg JL, Metter EJ (2007). Changes in leisure time physical activity and risk of all-cause mortality in men and women: the Baltimore Longitudinal Study of Aging. Prev Med.

[24] Grandes G, Sanchez A, Montoya I (2011). Two-year longitudinal analysis of a cluster randomized trial of physical activity promotion by general practitioners. PloS One.

[25] Sallis JF, Haskell WL, Wood PD (1985). Physical activity assessment methodology in the Five-City Project. Am J Epidemiol.

[26] Zuazagoitia A, Montoya I, Grandes G (2014). Reliability and validity of the 7-day Physical Activity Recall interview in a Spanish population. Eur J Sport Sci.

[27] Ferguson B (2014). ACSM’s Guidelines for Exercise Testing and Prescription 9th Ed. 2014. J Can Chiropr Assoc.

[28] Starfield B, Weiner J, Mumford L, Steinwachs D (1991). Ambulatory care groups: a categorization of diagnoses for research and management. Health Serv Res.

[29] Gómez Arnáiz A, Conde Martela A, Alberto Aguiar Bautista J (2001). [Diagnostic usefulness of Alcohol Use Disorders Identification Test (AUDIT) for detecting hazardous alcohol consumption in primary care settings]. [Article in Spanish]. Med Clin (Barc).

[30] Alvarez Dardet C, Alonso J, Domingo A, Regidor E (1995). [The measurement of social class in health sciences]. [Article in Spanish].

[31] Lipsky AM, Greenland S (2022). Causal directed acyclic graphs. JAMA.

[32] Rockhill B, Newman B, Weinberg C (1998). Use and misuse of population attributable fractions. Am J Public Health.

[33] Zhao M, Veeranki SP, Li S (2019). Beneficial associations of low and large doses of leisure time physical activity with all-cause, cardiovascular disease and cancer mortality: a national cohort study of 88,140 US adults. Br J Sports Med.

[34] Hupin D, Roche F, Gremeaux V (2015). Even a low-dose of moderate-to-vigorous physical activity reduces mortality by 22% in adults aged ≥60 years: a systematic review and meta-analysis. Br J Sports Med.

[35] Fuzeki E, Engeroff T, Banzer W (2017). Health benefits of light-intensity physical activity: a systematic review of accelerometer data of the National Health and Nutrition Examination Survey (NHANES). Sports Med.

[36] Wang Y, Nie J, Ferrari G (2021). Association of physical activity intensity with mortality: a national cohort study of 403 681 US adults. JAMA Intern Med.

[37] Koorts H, Eakin E, Estabrooks P (2018). Implementation and scale up of population physical activity interventions for clinical and community settings: the PRACTIS guide. Int J Behav Nutr Phys Act.

